# Supplementary material for: Rational Design of Potent α-Conotoxin PeIA Analogues with Non-Natural Amino Acids for the Inhibition of Human α9α10 Nicotinic Acetylcholine Receptors
Source: Mar Drugs. 2024 Feb 27;22(3):110. doi: 10.3390/md22030110 (PMC10971807; doi:10.3390/md22030110)
Supplement: Supplementary file 1 [file marinedrugs-22-00110-s001.zip › marinedrugs-2812787-supplementary.pdf]

Rational Design of Potent α-Conotoxin PeIA Analogues with Non-Natural Amino Acids for the Inhibition of Human α9α10 Nicotinic Acetylcholine Receptors

Tianmiao Li [1,2,†], Han-Shen Tae [3,†], Jiazhen Liang [1,2], Zixuan Zhang [1,2], Xiao Li [1,2], Tao Jiang [1,2], David J. Adams [3,*] and Rilei Yu [1,2,4,*]

1 Key Laboratory of Marine Drugs, Chinese Ministry of Education, School of Medicine and Pharmacy, Ocean University of China, 5 Yushan Road, Qingdao 266003, China; litianmiao99@163.com (T.L.); 17863973220@163.com (J.L.); zzx7212@stu.ouc.edu.cn (Z.Z.); 17854203983@163.com (X.L.); jiangtao@ouc.edu.cn (T.J.)
2 Laboratory for Marine Drugs and Bioproducts, Qingdao National Laboratory for Marine Science and Technology, Qingdao 266003, China
3 Molecular Horizons, Faculty of Science, Medicine and Health, University of Wollongong, Wollongong, NSW 2522, Australia; hstae@uow.edu.au
4 Innovation Center for Marine Drug Screening & Evaluation, Qingdao National Laboratory for Marine Science and Technology, Qingdao 266003, China
* Correspondence: djadams@uow.edu.au (D.J.A.); ryu@ouc.edu.cn (R.Y.)
† These authors contributed equally to this work.

Table S1. Pairwise interactions between PeIA (left) and PeIA[S4Dap, S9Dap] (right) at α9(+)α9(−) binding sites of hα9α10 nAChR.

PeIA residue	α9(+)α9(−)		α9(+)α9(−)		PeIA[S4Dap,S9Dap] residue
	α9(+)	α9(−)	α9(+)	α9(−)	
S4		D169		D166, S168, D169	Dap4
P6	W149		W149		P6
S9				Q34	Dap9
N11			N154		N11
H12	Y197		Y197		H12
P13		I59, L115		I59, L115	P13
E14		**R111**		**R111**	E14

Contacts between hα9α10 nAChR and PeIA/PeIA[S4Dap, S9Dap] are defined as van der Waals interactions if the distance between heavy atoms of them is between 2 and 4 Å. The electronic interactions are in bold.

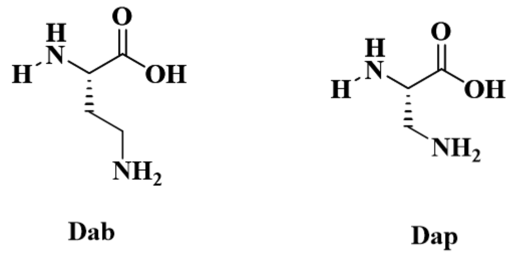

Figure S1. The structures of amino acids Dab-OH and Dap-OH. The non-proteinogenic amino acids Dab-OH and Dap-OH contain the same two and one methylene chain, respectively.

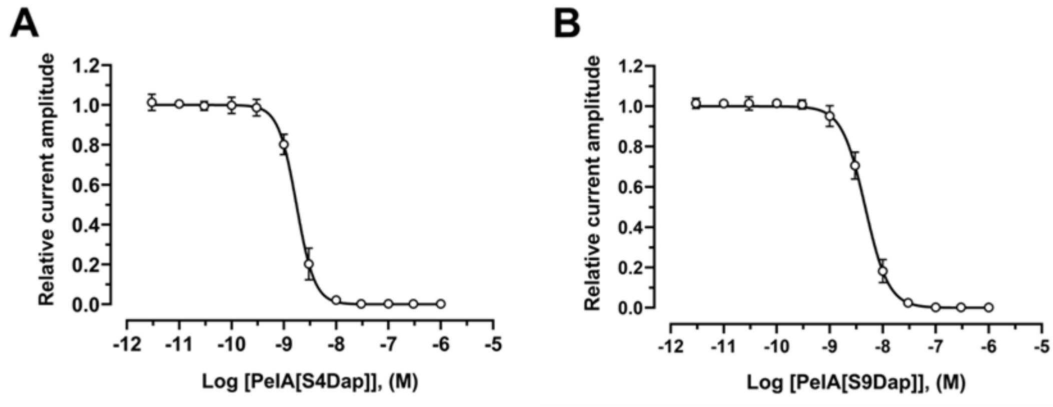

Figure S2. Concentration–response relationships of relative ACh-evoked current amplitude (mean ± SD, n = 7) mediated by hα9α10 nAChRs in the presence of (**A**) PeIA[S4Dap] and (**B**) PeIA[S9Dap] giving IC_{50}'s of 1.74 nM (1.67 – 1.82; 95% CI) and 4.67 nM (4.45 – 4.90; 95% CI), respectively. Whole-cell currents at hα9α10 were activated by 6 μM ACh.

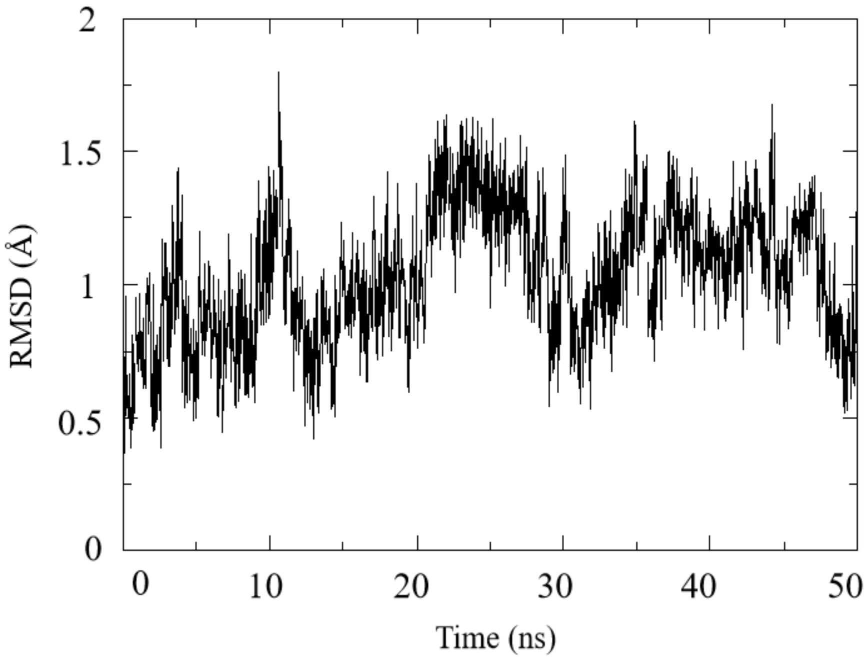

Figure S3. Evolution of root mean square deviation (RMSD) of PeIA.

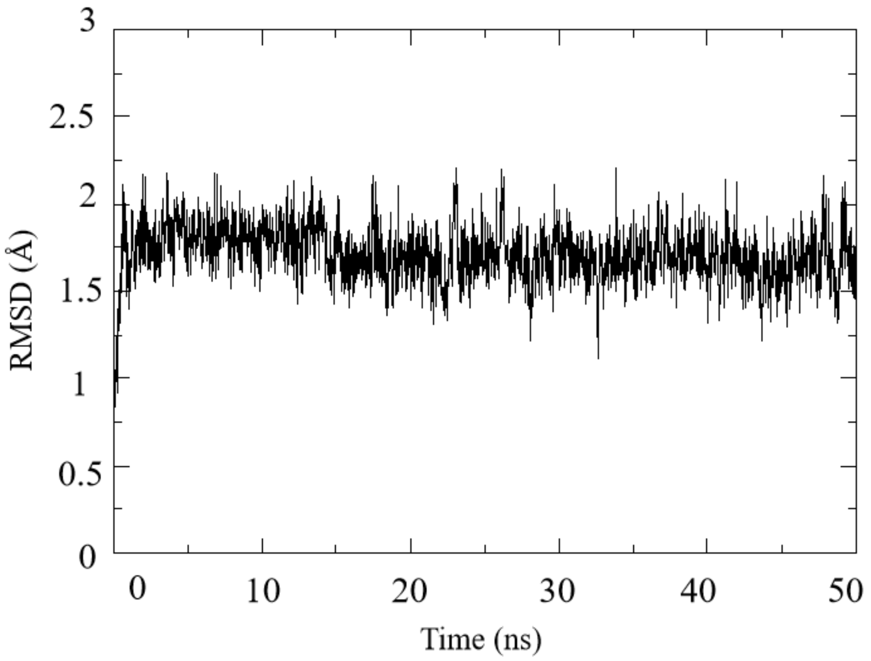

Figure S4. Evolution of root mean square deviation (RMSD) of PeIA[S4Dap, S9Dap].

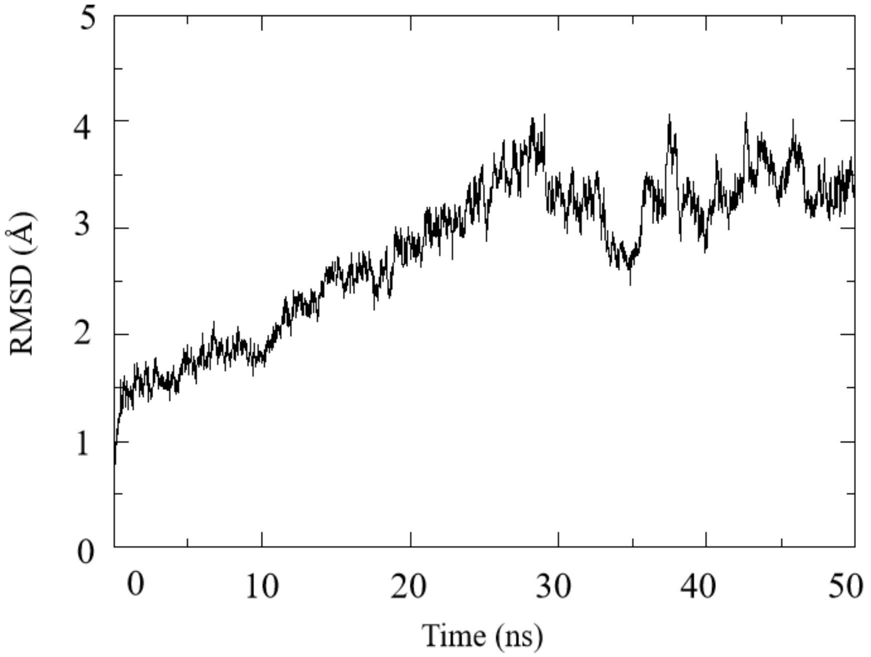

Figure S5. Evolution of root mean square deviation (RMSD) of PeIA bound to h$\alpha9\alpha10$ nAChR.

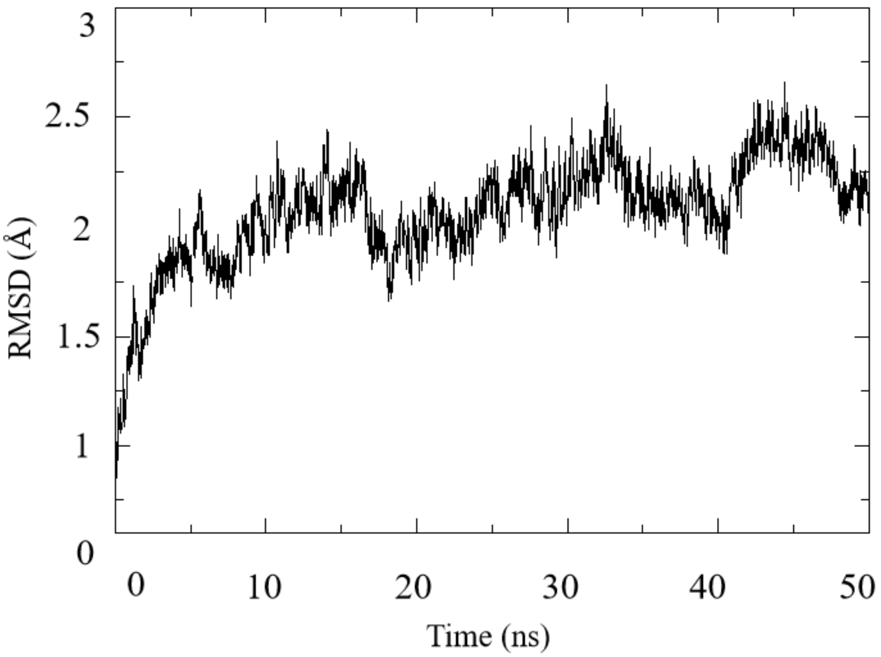

Figure S6. Evolution of root mean square deviation (RMSD) of PeIA[S4Dap, S9Dap] bound to hα9α10 nAChR.

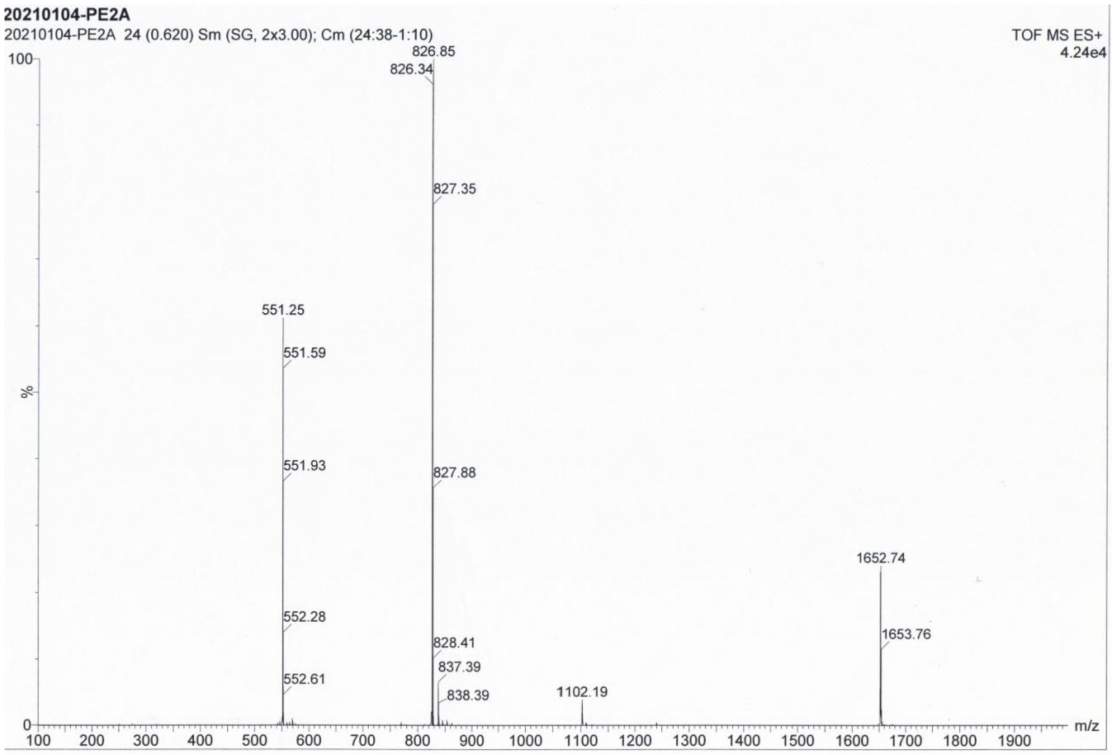

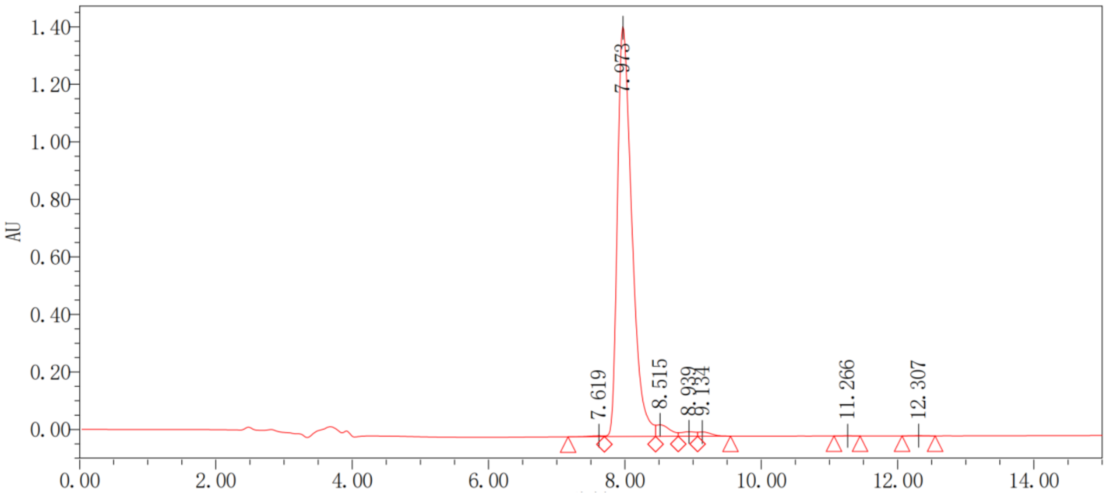

Figure S7. The ESI-MS and RP-HPLC of PeIA with a purity of 95.36%.

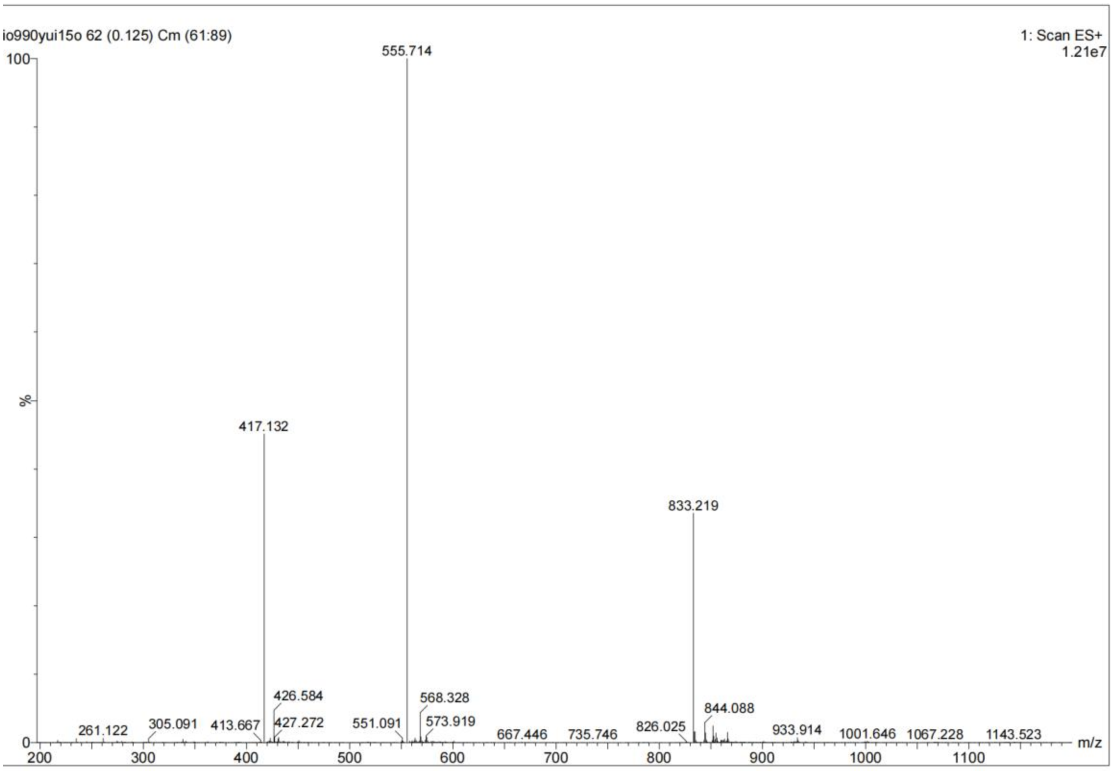

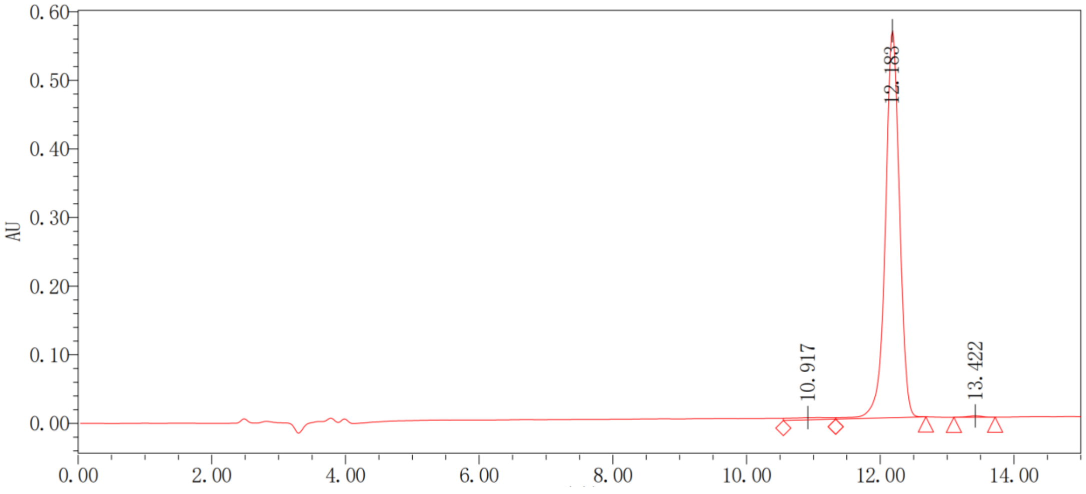

Figure S8. The ESI-MS and RP-HPLC of PeIA[S4Dab] with a purity of 99.67%.

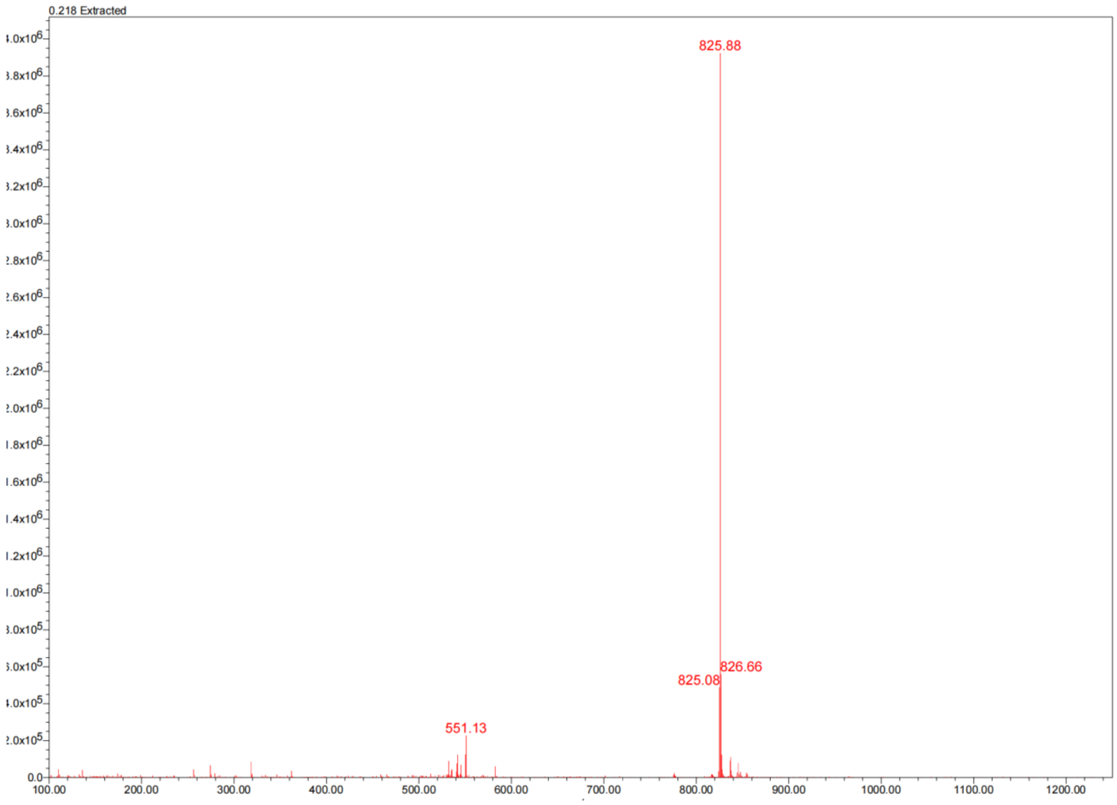

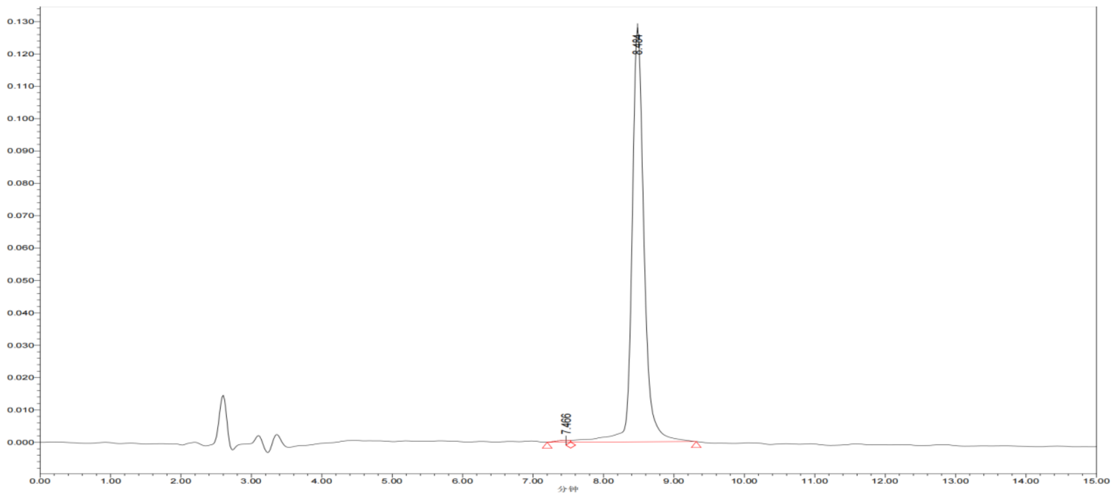

Figure S9. The ESI-MS and RP-HPLC of PeIA[S4Dap] with a purity of 99.53%.

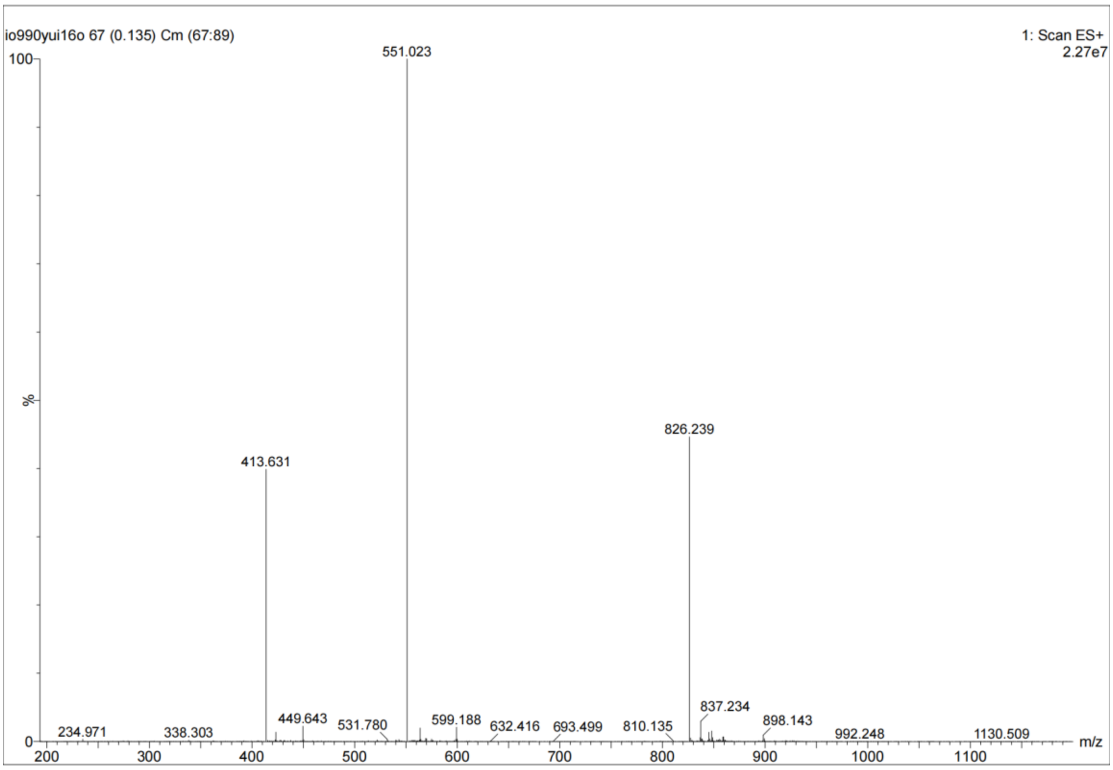

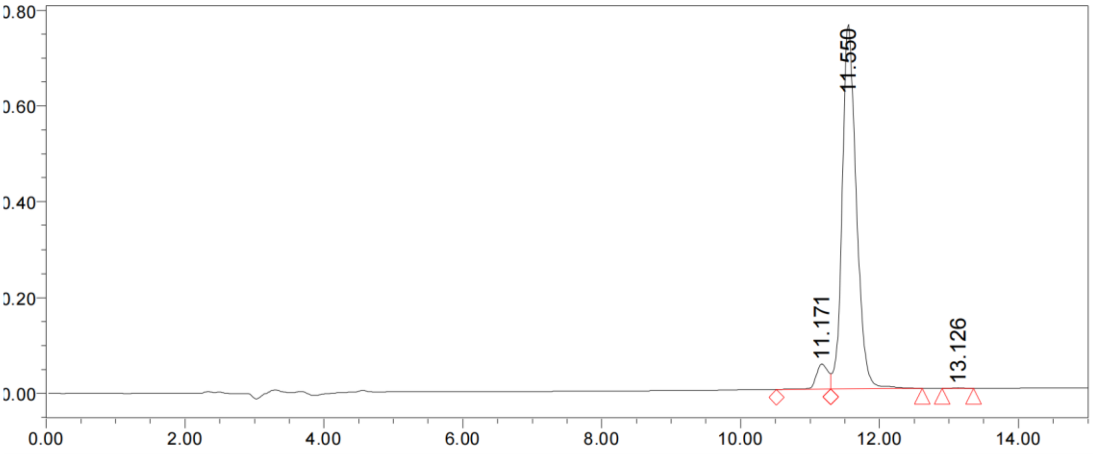

Figure S10. The ESI-MS and RP-HPLC of PeIA[S9Dap] with a purity of 94.68%.

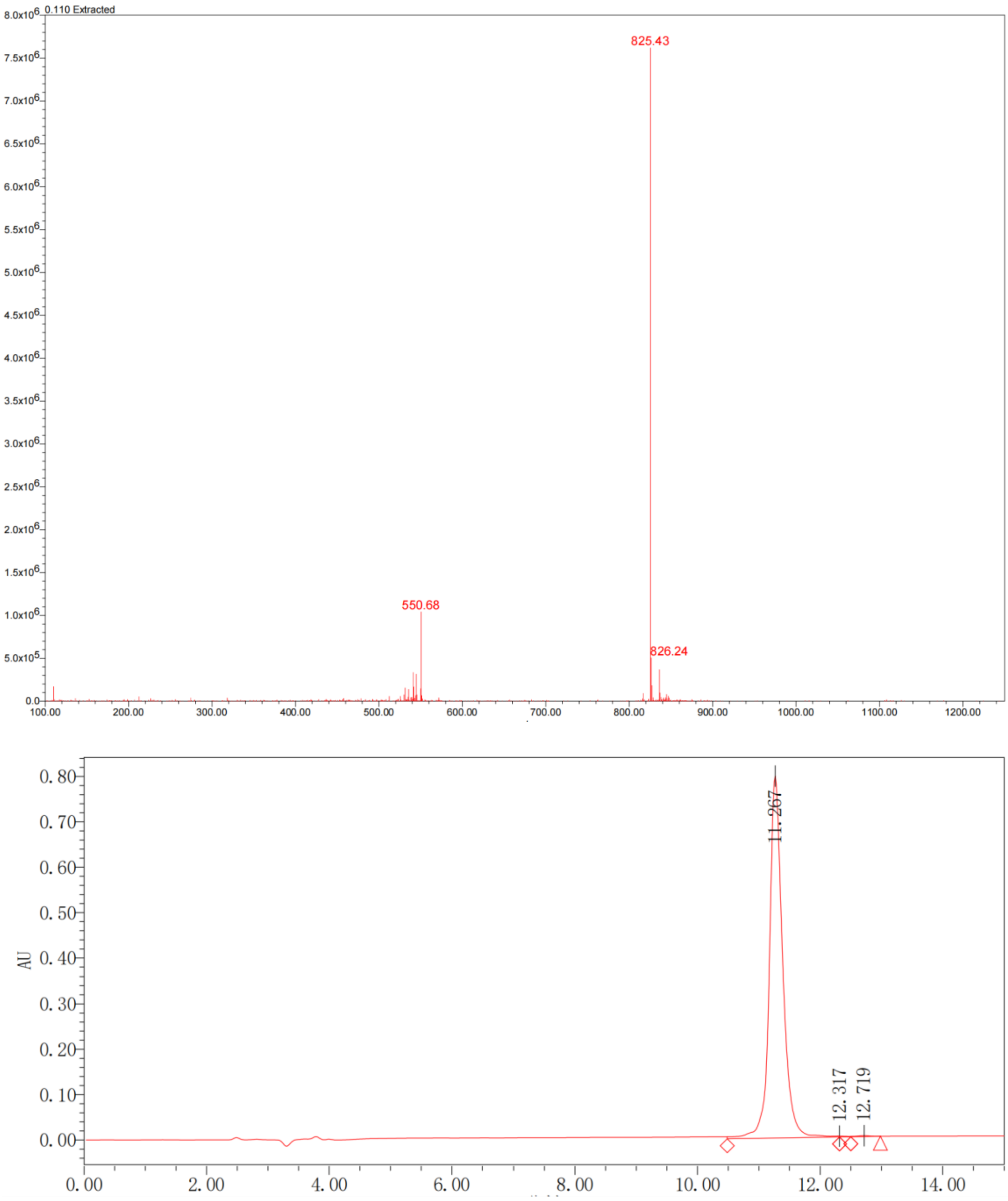

Figure S11. The ESI-MS and RP-HPLC of PeIA[S4Dap, S9Dap] with a purity of 99.28%.
